# Accelerometer-Based Human Fall Detection Using Convolutional Neural Networks

**DOI:** 10.3390/s19071644

**Published:** 2019-04-06

**Authors:** Guto Leoni Santos, Patricia Takako Endo, Kayo Henrique de Carvalho Monteiro, Elisson da Silva Rocha, Ivanovitch Silva, Theo Lynn

**Affiliations:** 1 Centro de Informática, Universidade Federal de Pernambuco, Recife 50670-901, Brazil; guto.leoni@gprt.ufpe.br; 2 Universidade de Pernambuco, Recife 50100-010, Brazil; khcm@ecomp.poli.br (K.H.d.C.M.); esr2@ecomp.poli.br (E.d.S.R.); 3 Business School, Dublin City University, Dublin 9, Ireland; theo.lynn@dcu.ie; 4 Universidade Federal do Rio Grande do Norte, Natal 59078-970, Brazil; ivan@imd.ufrn.br

**Keywords:** deep learning, human fall detection, sensor, accelerometer, convolutional neural networks

## Abstract

Human falls are a global public health issue resulting in over 37.3 million severe injuries and 646,000 deaths yearly. Falls result in direct financial cost to health systems and indirectly to society productivity. Unsurprisingly, human fall detection and prevention are a major focus of health research. In this article, we consider deep learning for fall detection in an IoT and fog computing environment. We propose a Convolutional Neural Network composed of three convolutional layers, two maxpool, and three fully-connected layers as our deep learning model. We evaluate its performance using three open data sets and against extant research. Our approach for resolving dimensionality and modelling simplicity issues is outlined. Accuracy, precision, sensitivity, specificity, and the Matthews Correlation Coefficient are used to evaluate performance. The best results are achieved when using data augmentation during the training process. The paper concludes with a discussion of challenges and future directions for research in this domain.

## 1. Introduction

Falls are a normal part of the human development life cycle. As children learn to stand, walk, climb, run and pursue other activities, falls occur. Similarly, as we age, falls also become part of our life experience. While most falls are of little consequence, the frequency and impact of falls increase dramatically with age. Falls are such a part of the human experience that we often underestimate the impact that they have on individuals and society. The World Health Organization (WHO) reports that falls cause over 37.3 million severe injuries and 646,000 deaths per annum. Falls are both a major health and economic issue worldwide. They place a significant burden on health systems, both in terms of in-patient and long-term care, but, in addition to the immediate direct physical effects, falls also can result in indirect psychological effects including reduction or avoidance of physical activity for fear of falling. From an economic perspective, clearly any increased burden on health system results has a direct financial cost to society; however, there is also an often forgotten cost in lost productivity.

Unsurprisingly, the frequency and impact of falls, the risk of death, healthcare costs, and lost societal and economic productivity have driven research and innovation in the area of fall detection. Earlier generations of fall detection systems were environmental sensor-based, which monitored the focal person in that location and collected relevant information on the movement of that person. These systems had inherent location-based limitations.

More recently, the evolution and widespread adoption of cloud computing, mobile technologies, and big data analytics combined with advances in low-cost sensing technologies have accelerated research in mobile healthcare monitoring as part of the so-called Internet of Things (IoT). Wearable devices for health monitoring are commonplace for children and adults of all ages; they may be dedicated wrist-worn devices, integrated in to smartphones, or a smartwatch. Wearable fall detection systems have significant advantages over previous generations of fixed-location fall detection systems. They are location-agnostic, monitor the focal person continuously, detect not only falls but also other pre-defined anomalous or predictive behaviour, and can alert third parties for intervention in advance or subsequent to a fall, thus remediating impact. While advances in cloud and mobile computing have enabled continuous monitoring, fall detection is empowered by advances in data science.

This article focuses on the fall detection and addresses calls for more research on the use of deep learning analysis in healthcare. We consider deep learning for fall detection in an IoT and fog computing environment. We propose a Convolutional Neural Network (CNN) model, which we label CNN-3B3Conv, and evaluate its performance (i) against extant research on fall detection using a Long Short-Term Memory (LSTM) approach, and (ii) using three different data sets. Accuracy, precision, sensitivity, specificity, and the Matthews Correlation Coefficient (MCC) are used to evaluate performance in experiments.

The rest of this paper is organized as follows. In the next section, we introduce deep learning, CNNs, LSTM, and related works. Then, we present our use case—a connected healthcare monitoring system for IoT-enabled human fall detection using fog computing and deep learning. This is followed by the results of experimental analysis. We conclude the article with a discussion of challenges and future avenues of research.

## 2. An Overview of Deep Learning

Traditional machine learning algorithms are limited by their ability to process raw data. Historically, it was necessary to extract data characteristics manually from data, requiring domain expertise to convert the raw data into a representation that the system could detect. In contrast, deep learning methods (a variation of neural networks) can automatically discover the representations needed for detection or classification from raw data without costly human intervention [1].

Neural networks are composed of many simple connected models called neurons. These neurons are typically arranged in sequential layers, where the input of a layer is the output of previous layer, with selected neurons that can influence the subsequent layers (feedforward) through triggering actions [2].

Neural networks are composed of three types of layers: input, output, and hidden layer. The input layer receives the raw data with the final layer classifying the data in to the desired categories [2]. A neural network can have one or more intermediate layers, referred to as hidden layers, have internal and adjustable parameters called weights. These weights, represented as matrices, map the input into output and are adjusted in the training phase [1,2].

Deep learning encompasses a wide variety of techniques. Each one deals with different classes of problems and data types such as images, audio, text, and video. CNN is a type of deep learning network designed to process data in multiple arrays. For example, signal data or text can be arranged as 1D arrays; audio spectrograms or images as 2D arrays; and video as 3D arrays [1].

### 2.1. Convolutional Neural Network (CNN)

**Figure 1:**
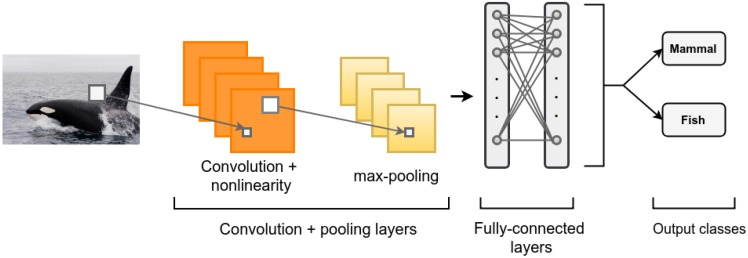
Example of a CNN for image classification.

Figure 1 provides an overview of a basic CNN for image classification. The CNN has two main layer types: convolutional and pooling. The convolutional layers generate a set of feature maps according to the number of pre-defined filters (or weights). These features are used to apply a convolution to the input data. The convolution is performed as a sliding window, where the feature convolves over a local region of the data and produces an output, which then becomes the input of the next layer [3]. Once the feature maps are computed, a nonlinear activation function is applied, such as ReLu, sigmoid, or tanh [4].

The pooling layers are used to merge semantically similar features (the output of the convolutional layer) into one output value [1]. The most common pooling technique used is maxpooling, where the maximum value from a sub-sampling region is used as the output value [3]. Finally, fully-connected layers are added after pooling layers to classify or make predictions from the input data [5,6].

### 2.2. Long Short-Term Memory (LSTM)

While CNNs are designed to deal with data arranged in multiple arrays, Recurrent Neural Networks (RNNs) were proposed to deal with time series. RNNs make use of a special neuron with recurrent connection. This connection acts as a memory, allowing the RNN to learn the temporal dynamicity of sequential data [7].

**Figure 2:**
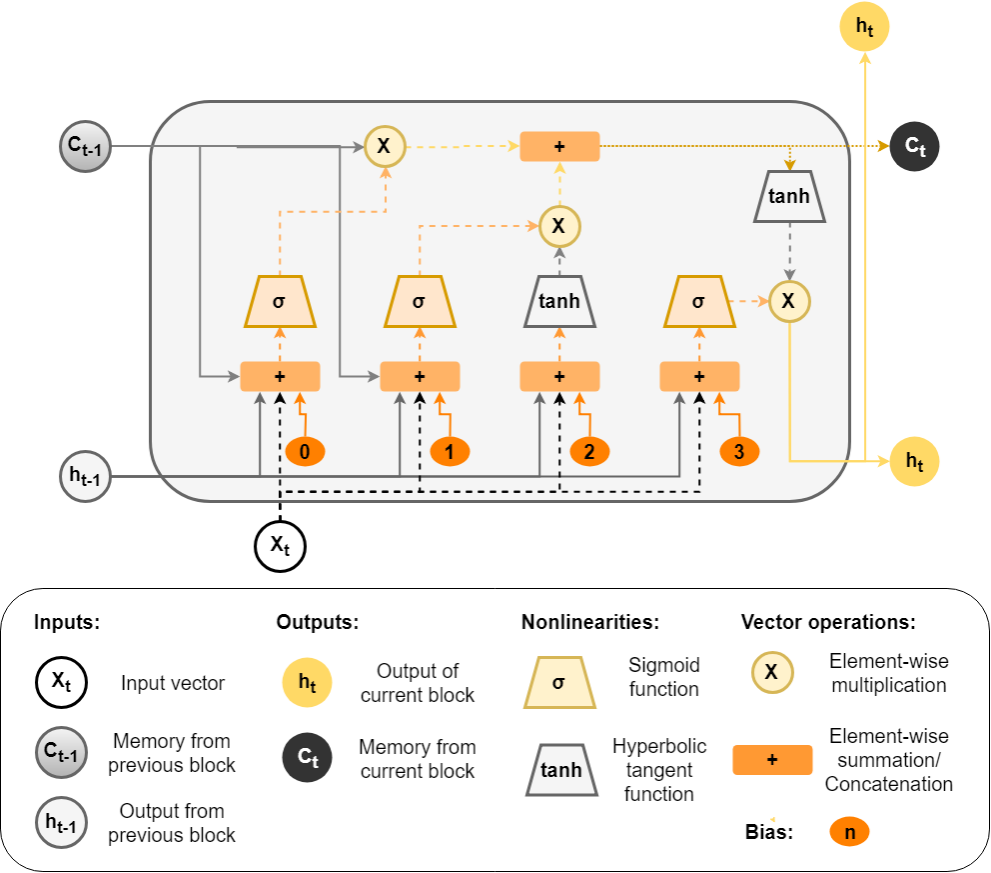
Example of an LSTM cell.

When dealing with long time dependencies, RNNs suffer from a problem called vanish gradient [8]. LSTM is a variation of traditional RNN to solve this issue. In LSTM networks, the recurrent neuron is replaced by a memory cell (see the Figure 2) and its operation is controlled by three gates: input gate, forget gate, and output gate. The output of these gates are based on component-wise multiplication of the input [7].

When the input gate is activated, the input data are accumulated in the cell and the forgot gate decides if the information is propagated forward or forgotten. This process is made through a sigmoid function, which outputs a value between zero (“completely forget”) and one (“completely keep”). Finally, the output gate controls the output cells according to the input data. Using these mechanisms to control the information flow through the cells, the gradient is trapped within the cell, preventing the vanish gradient problem [9].

### 2.3. Related Works: Deep Learning and Mobile-Enabled Fall Detection

A number of recent studies have been reported on human fall detection based on accelerometer data using deep learning networks.

In [10], a CNN model composed of three convolution layers and three pooling layers is proposed where the convolution kernel is adapted to the characteristics of the accelerometer data. A data set comprising 31,688 samples and featuring eight activities is used for analysis—falling, running, jumping, walking, walking, step walking, walking upstairs, and walking downstairs. The data was recorded using an Android-based smartphone embedded with an accelerometer sensor. The study compared Support Vector Machine (SVM) and Deep Belief Network (DBN) approaches with CNN; the CNN presented the best results with 93.8% accuracy.

In [11], three deep learning models for high risk fall prediction are proposed: a CNN, a Long Short-Term Memory (LSTM), and a combination of these two models (named ConvLSTM). Again, the data was collected through an accelerometer sensor and comprised 296 records of elderly people aged between 65 and 99 years. The Area Under the Curve (AUC) metric was used to compare the models. While both the LSTM and ConvLSTM models obtained better results than the CNN, the ConvLSTM model had a significantly faster execution time than the LSTM.

In [12], an RNN model, named LSTM-Acc and a variant LSTM-Acc Rot, are proposed. LSTM-Acc comprises two LSTM layers and two traditional feed-forward neural networks. To achieve greater model generalization and robustness, training data are augmented with one rotated version of the original measurements by a random angle about the *x*-, *y*- and *z*-axes. This second approach is named LSTM-Acc Rot. To compare results against extant research, namely, Acc + SVM-Depth [13] and UFT [14]), the UR Fall Detection (URFD) data set was used. The LSTM-Acc Rot obtained the best performance with an accuracy of 98.57%.

SmartFall is presented in [15], an Android application that collects accelerometer data through a smartwatch and uses deep learning for fall detection and prediction. Two traditional machine learning algorithms, SVM and Naive Bayes, are compared against a Gated Recurrent Units (GRU) model. The GRU model comprises three nodes at the input layer, a GRU layer, a fully connected layer, and a two-node softmax output layer. Three data sets were used to train and evaluate the models—FARSEEING (with real data), SmartWatch (with data collected from seven subjects wearing an MS Band watch), and Notch (with simulated data). The GRU model obtained the best result with 79% accuracy for the Notch data set.

In this study, we present a CNN model, named CNN-3B3Conv, that is less complex than the CNNs proposed by [10,11] in terms of number of layers and neurons, so that it would have less parameters to be updated during the training process, thereby resulting in a shorter training time. Three open data sets used by [12,15]—the URFD data set, SmartWatch data set, and Notch data set—were used to evaluate CNN-3B3Conv.

## 3. Experiment Use Case

**Figure 3:**
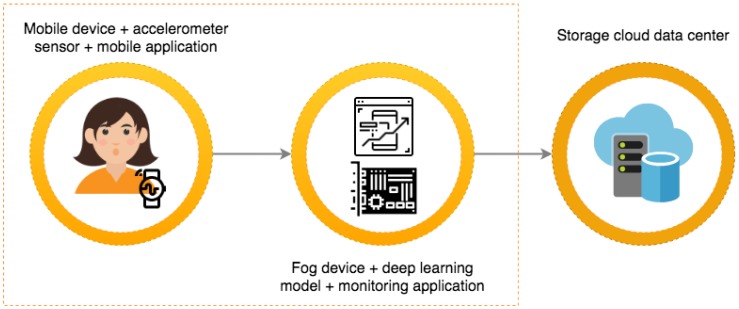
Simplified fall detection use case.

Figure 3 illustrates a use case for a IoT-enabled connected healthcare system for detecting human falls using an end device (embedded with an accelerometer sensor), a fog device, and deep learning.

A user can have one or more IoT devices—for example, a smartphone or smartwatch that collect accelerometer data. This data is used to feed a trained deep learning model deployed in a local fog device. The IoT devices communicate with the fog devices through wireless technologies, such as IEEE 802.11, Zigbee, and Bluetooth Low Energy [16]. In addition, the accelerometer data must be encoded using IoT communication protocols, such as Message Queue Telemetry Transport (MQTT) and Advanced Message Queuing Protocol (AMQP) [17].

Here, a fog device (such as Raspberry Pi) is used as an intermediary agent with three main tasks: (i) processing the accelerometer data (generated by the end user) using the trained deep learning model, (ii) sending the collected data to a cloud instance for long-term storage and further analysis, and (iii) hosting a monitoring application to generate notifications, when and if necessary i.e., when a fall is predicted or detected.

### IoT-Enabled Human Fall Detection

**Figure 4:**
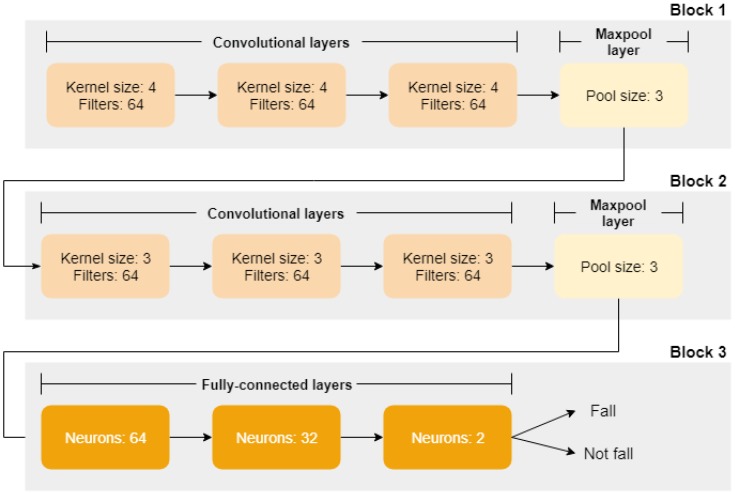
CNN-3B3Conv model to detect fall from accelerometer data.

The focus of this study is a deep learning model deployed at the fog device used to process the accelerometer data generated by the end user. We propose a CNN to extract relevant features from the data and detect if a fall occurred or not. Figure 4 presents the architecture of our CNN model, named CNN-3B3Conv.

Block 1 comprises a set of three sequential convolutional layers followed by one pooling layer. As we are considering sequential data, the model is designed with a one-dimensional convolution in the convolutional layers. For each convolutional layer, we configured a Rectified Linear Unit (ReLU) as the activation function and L2 regularization with α=0.01 to reduce the overfitting. Moreover, we used 64 filters and a kernel size equal to four. After these three convolutional layers, there is a pooling layer used to merge semantically similar features, reducing the data dimensionality. We configured maxpooling with a pool size equal to three, and dropout with probability of 35% to reduce overfitting (These values were chosen empirically).

Similarly, Block 2 comprises another set of three sequential convolutional layers and one pooling layer. However, the unique difference is the kernel size, which we configured to three.

Finally, Block 3 comprises three fully-connected layers with 64, 32 and two neurons, respectively. The last layer has only two neurons reflecting the output of the model: whether the event is a “fall” or “not fall”.

We use the logarithm of the hyperbolic cosine as a loss function as defined by Keras (https://keras.io/losses/logcosh). To minimize the error estimated by this function, we use Stochastic Gradient Descent [18] with a learning rate equal to 0.0107, the momentum equal to 0.999, and learning rate decay over each update equals to $1\times 10^{-6}$; we use Nesterov momentum [19]. These parameters were chosen empirically, and we trained the model over 20 epochs.

## 4. Evaluation

We take two approaches to evaluate our CNN model. Firstly, compare CNN-3B3Conv’s performance against the models proposed in [12] (Experiment 1). Secondly, we compare CNN-3B3Conv’s performance when using different data sets (Experiment 2).

### 4.1. Methodology

#### 4.1.1. Experiment 1

Experiment 1 seeks to compare CNN-3B3Conv’s results against the models proposed by [12]. As discussed in Section 2, authors in [12] used the URFD data set to train and evaluate their initial model, LTSM-Acc. However, as the data set has a limited number of samples, a data augmentation (DA) technique was applied to improve the model generalization and performance; this variation was named LTSM-Acc Rot. To allow like-for-like comparison, we also applied the DA technique to train our CNN-3B3Conv.

DA is a technique that applies one or more deformations through the limited data to generate new samples, without changing the semantic meaning of the labeled data [20]. For example, in the computer vision context, a rotated, translated or mirrored image of a cat would still be a coherent image of a cat, thus it is possible apply these deformations in order to produce additional training data keeping the semantic validity of the label. In our case, the DA presented in [12] consists of random rotations in the accelerometer data to generate new samples. Consider the acceleration vector a(t)=[ax(t),ay(t),az(t)] at time *t*, containing the acceleration along the *x*-, *y*- and *z*-axes; then, the new vector $a_{r}\left(t\right)$ is generated by rotating a(t) by α, β, and γ radians about the *x*-, *y*- and *z*-axes, respectively.

The URFD is composed of 30 samples of falls and 40 samples of activities of daily living (ADL). As the number of new samples generated is not described in [12], we generated 1800 and 3200 new samples of falls and ADLs respectively, totalling 5000 new samples. We split 80% of augmented data set for training and 20% for testing, totalling 4000 and 1000 samples, respectively. After the training, we normalized the data set; values are between zero and one in order to reduce the magnitude of input data that can influence the model performance [21].

We set up the input layer of CNN-3B3Conv to read input with a shape equal to 500×3 (where 500 is the number of lines of each sample, and three are the dimensions *x*, *y*, and *z*).

#### 4.1.2. Experiment 2

In Experiment 2, we compare CNN-3B3Conv’s performance using different data sets with and without the DA technique. For this experiment, we consider the SmartWatch and Notch data sets provided by [15]. We could not compare our model against the model presented by [15] because our CNN-3B3Conv processes the data sets input in a different way, so the comparison would not be scientifically robust.

In this experiment, CNN-3B3Conv was adapted to allow it to read the SmartWatch data set and therefore resolve dimensionality issues (see the discussion in Section 5). As a result, we have two variants of the our CNN model presented in Figure 4:

- **CNN-1Conv**: CNN with two blocks, Block 1 and Block 3 where Block 1 has only one convolutional layer and one maxpool layer; and
- **CNN-3Conv**: CNN with two blocks, Block 1 and Block 3 where Block 1 uses all layers (three convolutional layers and one maxpool layer).

The SmartWatch data set was divided into samples of 25×3 (the fall event duration is 25 lines) and the Notch data set into samples of 118×3 (the longest duration of a fall event in this data set is 118). The SmartWatch data set was originally comprised 182 falls and 1088 ADL samples. After data augmentation, the data set increased to 1092 falls and 1088 ADL sequences. ADL events in this data set consist of jogging, sitting down, throwing an object, and waving their hands. Similarly, the Notch data set originally comprised 106 falls and 568 ADLs; after data augmentation, this increased to 530 falls and 568 ADLs. We divided both the SmartWatch and Notch data sets with 80% of the data for training and 20% for testing.

### 4.2. Metrics

The following metrics are used to evaluate the models: accuracy, sensitivity, specificity and MCC. Accuracy is the rate of correct classification and is calculated as:

(1) $$\mathrm{acccuracy}=\frac{\mathrm{TP}\;+\;\mathrm{TN}}{\mathrm{TP}\;+\;\mathrm{FP}\;+\;\mathrm{TN}\;+\;\mathrm{FN}},$$

where TP is the true positive rate, TN is the true negative rate, FP is the false positive rate, and FN is the false negative rate.

A precision score of 100% for binary classification means that every item labeled as belonging to the positive class does indeed belong to the positive class and is calculated as:

(2) $$\mathrm{precision}=\frac{\mathrm{TP}}{\mathrm{TP}\;+\;\mathrm{FP}}.$$

Sensitivity (or true positive rate) is the probability of a positive test result amongst those having the target condition and is calculated as:

(3) $$\mathrm{sensitivity}=\frac{\mathrm{TP}}{\mathrm{TP}\;+\;\mathrm{FN}}.$$

Specificity (or true negative rate) is the probability of a negative test result amongst those without the target condition and is calculated using the following equation:

(4) $$\mathrm{specificity}=\frac{\mathrm{TN}}{\mathrm{TN}\;+\;\mathrm{FP}}.$$

MCC is a metric used widely to measure performance when dealing with imbalanced data and is defined in terms of TP, TN, FP and FN [22]:

(5) $$\mathrm{MCC}=\frac{\left(\mathrm{TP}\times \mathrm{TN})-(\mathrm{FP}\times \mathrm{FN}\right)}{\sqrt{\left(\mathrm{TP}+\mathrm{FP})\times (\mathrm{TP}+\mathrm{FN})\times (\mathrm{TN}+\mathrm{FP})\times (\mathrm{TN}+\mathrm{FN}\right)}}.$$

### 4.3. Results

In this section, we present the results of our models: CNN3B3Conv, and its two variations, CNN-1Conv and CNN-3Conv (see Section 4.1.2).

#### 4.3.1. Experiment 1

As stated in Section 4.1.1, the main goal of the Experiment 1 is to compare CNN-3B3Conv’s results against the models proposed by [12]. Table 1 presents the accuracy, precision, sensitivity, and specificity results of our CNN model and the models LSTM-Acc and LSTM-Acc Rot ([12]) with and without the data augmentation technique.

Our CNN-3B3Conv model with DA achieves the best results regarding accuracy and sensitivity compared to proposals presented by [12]. The CNN-3B3Conv model and the LSTM Acc Rot (both with DA) present the same precision and specificity results (100% for both metrics), meaning that they do not produce false positive results i.e., they do not detect a fall when no fall has occurred.

On the other hand, our CNN-3BN3Conv model without DA presented the worst results (comparing against all models) due to the limited number of samples for training the model, confirming the improvement when using a DA technique.

#### 4.3.2. Experiment 2

As stated in Section 4.1.2, experiment 2 seeks to compare the CNN-3B3Conv’s performance using two different data sets (SmartWatch and Notch) with and without the DA technique. Table 2 shows the comparison results regarding the SmartWatch (using both variants of our CNN model, CNN-1Conv and CNN-3Conv) and Notch data sets (using our original CNN model) with and without the DA technique applied during the training process.

The CNN-1Conv presents better results than the CNN-3Conv for all metrics, independently of whether DA is applied or not. This is an interesting result because, when one increases the model complexity (adding more layers and blocks), overffiting also increases. As in this case, simple models can be more accurate.

In summary, when using data augmentation, our CNN models present better performance. The exceptions are: the CNN-1Conv with and without data augmentation achieves 100% precision and specificity; and the CNN-3Conv without data augmentation presents better specificity (99.54%) than with data augmentation (98.53%). For the MCC metric, the CNN-1Conv with data augmentation presents the best performance, i.e., 0.9968, when using the SmartWatch data set.

Regarding the Notch data set, the results are interesting. When DA is used, our CNN-3B3Connv model achieves the best precision, sensitivity and MCC results. In contrast, without DA, the CNN presents the best accuracy and specificity results. As MCC integrates all these metrics, we can state that the CNN-3B3Conv with DA achieves the best results for the Notch data set (0.6270), while the same CNN3B3Conv but without DA achieves the worst result for the same data set.

## 5. Research Challenges and Opportunities

The results from this study highlight a number of challenges and future directions for fall detection research generally and deep learning specifically.

### 5.1. Deep Learning Techniques

In this study, we explore one deep learning technique and some different configurations. CNN and LSTM are two of the most common approaches in fall detection; however, they are not the only ones. Deep Belief Networks, Restricted Boltzmann Machines, Autoencoders, Time Delay Neural Networks, Multi-Layer Perceptrons, and Gated Recurrent Units have all been used for fall detection.

Further combinatory and comparative experimentation is needed using the range of deep learning techniques available. While our study used only CNN technique and combination of this, our results were promising and suggest improved performance through experimentation with configuration and through the use of data augmentation. Consequently, further experimentation with different techniques for both learning and pre-training may provide improved results.

### 5.2. Fall Detection Data

This study focused on one type of fall detection system—a wearable-sensor based system using accelerometer data for fall detection. While use is made of three data sets representing different edge devices, the type of data being used is relatively homogeneous. We do not explore a second category of fall detection system—environmental sensor-based systems. Such systems are installed in the environment in which focal persons interact and then collect relevant information about their movement through a wide range of sensors including cameras, infrared sensors, acoustic sensors, and piezoelectric sensors amongst others.

The combination of multi-dimensional data sets on fall detection, or more ideally the same set of focal persons, offers significant opportunities for research and model performance improvement in real-world settings. Notwithstanding this opportunity, this presents significant feature extraction challenges.

### 5.3. Adequate Research Validation

Despite a substantial volume of research on fall detection and an increasing number of scientific publications on the use of neuronal techniques, including deep learning, significant challenges remain in adequate validation of experimental research. Extant research is largely based on distinct experiments with proprietary data sets with limited volume, variety, velocity and verisimilitude. Indeed, such issues were encountered in this study and discussed in Section 4. Few are operationalized in live environments and, as such, their performance in real-world conditions and context is largely unknown. As such, comparative evaluation in the literature is not only relatively rare but often not possible due to restricted access to software, data, and the technical minutiae of both.

Fall detection is an e-health use case and as such requires a more coordinated and rigorous approach to evaluation that supports reproducibility of results. Large, standardized open data sets for falls and other ADLs would be a significant step in the right direction, since, as highlighted in [23], data sets based on accelerometer and gyroscope are even scarce, making it difficult to develop such deep learning solutions based on this kind of data.

### 5.4. Computational Performance in the Internet of Medical Things

Gatouillat et al. [24] define the Internet of Medical Things (IoMT) as the interconnection of medical-grade devices with broader health care infrastructures, connecting personal medical devices with each other and with health care providers—whether hospitals, medical researchers, or private companies. The IoMT requires higher levels of reliability, safety and security than traditional systems due to the criticality of such systems, the impact of adverse outcomes, and the sensitivity of personal health information [24]. The IoMT is typically configured along a cloud-to-thing continuum comprising cloud computing, internet-connected gateways, and a sensor to collect data. Our study assumes a fall detection system operating in such an IoMT architecture. Notwithstanding the contributions of our study, such IoMT use cases present significant challenges from a computational performance perspective. Real-world fall detection requires near real-time computational performance and high levels of availability. Further research is required with regard to the performance and scalability of proposed deep learning models in real-world contexts in a variety of scenarios including edge, mist, fog, cloud computing scenarios and the impact of network latency and/or unavailability.

## 6. Conclusions

In this article, we consider deep learning for fall detection in an IoT and fog computing environment. We evaluate the performance of a CNN (i) against two other approaches used in extant research and (ii) using three different data sets. Accuracy, precision, sensitivity, specificity, and the Matthews Correlation Coefficient were used to evaluate performance in experiments.

Experimental results suggest that the proposed CNN model, CNN-3B3Conv, using data augmentation presents better results for fall detection compared to LTSM-Acc and LTSM-Acc Rot [12]. Similar to [12], we could demonstrate that applying the data augmentation technique improves the deep learning performance, both in terms of accuracy and precision. Dimensionality issues hampered the use of multiple data sets for evaluation, highlighting the need for large standardised data sets for comparative research validation. Notwithstanding this, once the CNN model was modified, performance was improved albeit with a simpler CNN model, the CNN-1Conv.

As future work, we plan to evaluate other deep learning approaches to detect human falls and other types of human activities, as well as making use of other public data sets [25] including data sets comprising multiple sensors (multimodal) located at different parts on the human body as per [26,27]. We also plan to improve our model to detect multi-class events and thereby distinguish different activities (e.g., walking, running, and jumping) and different fall types (e.g., falling forward, falling backwards, and falling sideways). In addition, we plan to investigate the impact of computing paradigm—for example, edge, fog and cloud computing on overall system performance.

## Figures and Tables

**Table 1:** Fall detection results (in %) when using the URFD data set.

	CNN-3B3Conv without DA	CNN-3B3Conv with DA	LSTM Acc [12]	LSTM Acc Rot [12]
Accuracy	85.71	**99.86**	95.71	98.57
Precision	83.33	**100.00**	95.00	**100.00**
Sensitivity	83.33	**99.72**	96.67	96.67
Specificity	87.50	**100.00**	95.00	**100.00**

**Table 2:** Fall detection results (in %) when using the SmartWatch and Notch data sets.

Data Set Model	SmartWatch CNN-1Conv	SmartWatch CNN-3Conv	Notch CNN-3B3Conv	SmartWatch CNN-1Conv	SmartWatch CNN-3Conv	Notch CNN-3B3Conv
	**without Data Augmentation**			**with Data Augmentation**		
Accuracy	99.13	98.43	**86.76**	**99.92**	98.43	79.55
Precision	**100.00**	97.09	83.33	**100.00**	91.75	**95.52**
Sensitivity	93.96	91.76	22.73	**99.45**	97.80	**60.38**
Specificity	**100.00**	99.54	**99.12**	**100.00**	98.53	97.37
MCC	0.9644	0.9349	0.3918	**0.9968**	0.9382	**0.6270**

## Abbreviations

The following abbreviations are used in this manuscript:

WHO	The World Health Organization
IoT	Internet of Things
CNN	Convolutional Neural Network
LSTM	Long Short-Term Memory
MCC	Matthews Correlation Coefficient
RNN	Recurrent Neural Networks
SVM	Support Vector Machine
DBN	Deep Belief Network
AUC	Area Under the Curve
URFD	UR Fall Detection
GRU	Gated Recurrent Units
MQTT	Message Queue Telemetry Transport
AMQP	Advanced Message Queuing Protocol
ReLU	Rectified Linear Unit
ADL	Activities of Daily Living
TP	True Positive
TN	True Negative
FP	False Positive
FN	False Negative
IoMT	Internet of Medical Things
